# Bis(2-methyl-1*H*-imidazole-κ*N* ^3^)silver(I) nitrate dihydrate

**DOI:** 10.1107/S1600536809045838

**Published:** 2009-11-07

**Authors:** Fang-Di Cong, Feng-Yang Yu, Zhen Wei, Seik Weng Ng

**Affiliations:** a Department of Basic Science, Tianjin Agricultural University, Tjianjin 300384, People’s Republic of China; b Department of Chemistry, University of Malaya, 50603 Kuala Lumpur, Malaysia

## Abstract

The Ag^I^ atom in the salt, [Ag(C_4_H_6_N_2_)_2_]NO_3_·2H_2_O, shows a nearly linear coordination [N—Ag—N = 178.26 (7)°]. The cation, anion and water mol­ecules are linked by N—H⋯O and O—H⋯O hydrogen bonds into a layer motif extending parallel to (101).

## Related literature

For the crystal structure of [Ag(C_4_H_6_N_2_)_2_][NO_3_]·CH_3_OH, see: Liu *et al.* (2006 ▶).
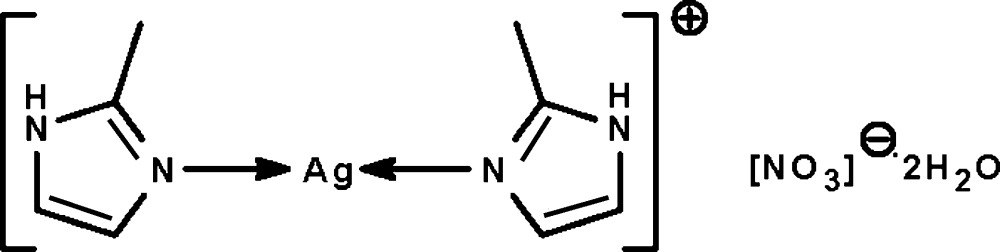


## Experimental

### 

#### Crystal data

[Ag(C_4_H_6_N_2_)_2_]NO_3_·2H_2_O
*M* *_r_* = 370.13
Monoclinic, 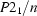

*a* = 6.8001 (4) Å
*b* = 17.0196 (9) Å
*c* = 12.1453 (7) Å
β = 101.691 (1)°
*V* = 1376.48 (13) Å^3^
*Z* = 4
Mo *K*α radiation
μ = 1.49 mm^−1^
*T* = 295 K
0.21 × 0.19 × 0.17 mm

#### Data collection

Bruker APEX2 diffractometer
Absorption correction: multi-scan (*SADABS*; Sheldrick, 1996 ▶) *T*_min_ = 0.745, *T*_max_ = 0.786
7483 measured reflections
2721 independent reflections
2083 reflections with *I* > 2σ(*I*)
*R*_int_ = 0.020

#### Refinement

*R*[*F* ^2^ > 2σ(*F* ^2^)] = 0.024
*wR*(*F* ^2^) = 0.073
*S* = 0.99
2721 reflections
198 parameters
6 restraints
H atoms treated by a mixture of independent and constrained refinement
Δρ_max_ = 0.54 e Å^−3^
Δρ_min_ = −0.34 e Å^−3^

### 

Data collection: *APEX2* (Bruker, 2007 ▶); cell refinement: *SAINT* (Bruker, 2007 ▶); data reduction: *SAINT*; program(s) used to solve structure: *SHELXS97* (Sheldrick, 2008 ▶); program(s) used to refine structure: *SHELXL97* (Sheldrick, 2008 ▶); molecular graphics: *X-SEED* (Barbour, 2001 ▶); software used to prepare material for publication: *publCIF* (Westrip, 2009 ▶).

## Supplementary Material

Crystal structure: contains datablocks global, I. DOI: 10.1107/S1600536809045838/bt5122sup1.cif

Structure factors: contains datablocks I. DOI: 10.1107/S1600536809045838/bt5122Isup2.hkl

Additional supplementary materials:  crystallographic information; 3D view; checkCIF report

## Figures and Tables

**Table 1:** Hydrogen-bond geometry (Å, °)

*D*—H⋯*A*	*D*—H	H⋯*A*	*D*⋯*A*	*D*—H⋯*A*
N2—H2⋯O1*w*	0.86 (1)	1.99 (1)	2.838 (3)	169 (3)
N4—H4⋯O1*w* ^i^	0.84 (1)	1.99 (1)	2.837 (3)	178 (3)
O1*w*—H11⋯O2*w*	0.85 (1)	1.89 (1)	2.726 (3)	170 (4)
O1*w*—H12⋯O1	0.85 (1)	1.99 (1)	2.826 (3)	171 (3)
O2*w*—H21⋯O1^ii^	0.84 (1)	2.02 (1)	2.867 (3)	179 (4)
O2*w*—H22⋯O2^iii^	0.84 (1)	2.15 (2)	2.955 (3)	159 (3)

Symmetry codes: (i) 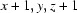; (ii) 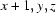; (iii) 
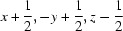
.

**Fig. 1.**
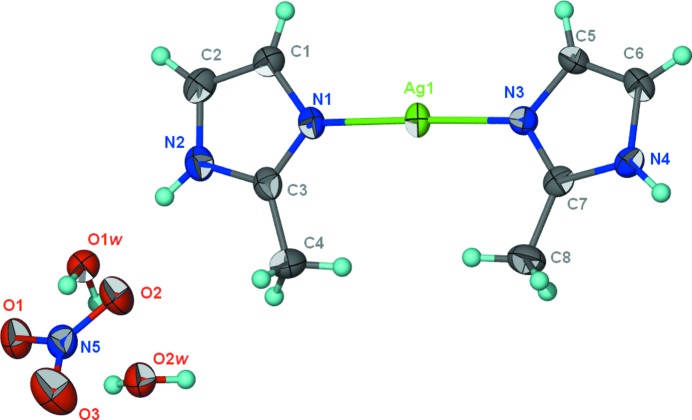
Anisotropic displacement ellipsoid plot (Barbour, 2001) of [Ag(C4H6N2)2][NO3].2H2O at the 70% probability level; hydrogen atoms are drawn as spheres of arbitrary radius.

## supplementary crystallographic information

### Experimental

Silver nitrate (0.5 mmol, 0.085 g) and 2-methyl-1*H*-imidazole (0.5 mmol, 0.041 g) in water (15 ml) were heated in a Parr bomb at 433 K for three days. Crystals of the adduct were isolated from the cool mixture in 30% yield.

### Refinement

Carbon-bound H-atoms were placed in calculated positions (C—H 0.93–0.97 Å) and were included in the refinement in the riding model approximation, with *U*(H) set to 1.2–1.5*U*(C). The amino and water H atoms were located in a difference Fourier map, and were refined with a distance restraint of N–H = O–H = 0.85±0.01 Å; their displacement parameters were refined.

The final difference Fourier map had a peak that was displaced by 0.5 along *y* relative to Ag1. Thus, for the reflections with k odd a scale factor was refined to 1.035 (2) with respect to the reflections with k even. Although the refinement was not significantly improved, the final difference Fourier map now did not have any large peaks.

### Crystal data

**Table:**

[Ag(C4H6N_2_)_2_]NO_3_·2H_2_O	*F*(000) = 744
*M**_r_* = 370.13	*D*_x_ = 1.786 Mg m^−^^3^
Monoclinic, *P*2_1_/*n*	Mo *K*α radiation, λ = 0.71073 Å
Hall symbol: -P 2yn	Cell parameters from 3827 reflections
*a* = 6.8001 (4) Å	θ = 2.9–26.1°
*b* = 17.0196 (9) Å	µ = 1.49 mm^−^^1^
*c* = 12.1453 (7) Å	*T* = 295 K
β = 101.691 (1)°	Block, colorless
*V* = 1376.48 (13) Å^3^	0.21 × 0.19 × 0.17 mm
*Z* = 4	

### Data collection

**Table:**

Bruker APEX2 diffractometer	2721 independent reflections
Radiation source: fine-focus sealed tube	2083 reflections with *I* > 2σ(*I*)
graphite	*R*_int_ = 0.020
φ and ω scans	θ_max_ = 26.1°, θ_min_ = 2.1°
Absorption correction: multi-scan (*SADABS*; Sheldrick, 1996)	*h* = −4→8
*T*_min_ = 0.745, *T*_max_ = 0.786	*k* = −19→21
7483 measured reflections	*l* = −15→14

### Refinement

**Table:**

Refinement on *F*^2^	Primary atom site location: structure-invariant direct methods
Least-squares matrix: full	Secondary atom site location: difference Fourier map
*R*[*F*^2^ > 2σ(*F*^2^)] = 0.024	Hydrogen site location: inferred from neighbouring sites
*wR*(*F*^2^) = 0.073	H atoms treated by a mixture of independent and constrained refinement
*S* = 0.99	*w* = 1/[σ^2^(*F*_o_^2^) + (0.0441*P*)^2^ + 0.0379*P*] where *P* = (*F*_o_^2^ + 2*F*_c_^2^)/3
2721 reflections	(Δ/σ)_max_ = 0.001
198 parameters	Δρ_max_ = 0.54 e Å^−^^3^
6 restraints	Δρ_min_ = −0.33 e Å^−^^3^

### Fractional atomic coordinates and isotropic or equivalent isotropic displacement parameters (Å^2^)

**Table:**

	*x*	*y*	*z*	*U*_iso_*/*U*_eq_	
Ag1	0.63346 (3)	0.501978 (10)	0.882866 (16)	0.02240 (9)	
O1	−0.1909 (3)	0.26078 (11)	0.44924 (15)	0.0315 (5)	
O2	−0.0592 (3)	0.30353 (12)	0.61645 (17)	0.0404 (5)	
O3	−0.2399 (3)	0.19831 (13)	0.59642 (18)	0.0458 (6)	
O1w	0.1172 (3)	0.35094 (12)	0.38606 (16)	0.0266 (4)	
O2w	0.3941 (3)	0.24560 (11)	0.34310 (17)	0.0292 (4)	
N1	0.4754 (4)	0.49985 (11)	0.7163 (2)	0.0217 (5)	
N2	0.2877 (3)	0.45702 (14)	0.55959 (19)	0.0257 (5)	
H2	0.228 (4)	0.4222 (13)	0.514 (2)	0.043 (9)*	
N3	0.7904 (3)	0.50045 (10)	1.0498 (2)	0.0201 (5)	
N4	0.9606 (3)	0.45734 (13)	1.21030 (19)	0.0219 (5)	
H4	1.010 (4)	0.4257 (12)	1.2620 (16)	0.025 (8)*	
N5	−0.1639 (3)	0.25410 (13)	0.55457 (19)	0.0275 (5)	
C1	0.3998 (4)	0.56352 (16)	0.6500 (2)	0.0242 (6)	
H1	0.4245	0.6160	0.6694	0.029*	
C2	0.2853 (4)	0.53782 (17)	0.5535 (2)	0.0267 (6)	
H2A	0.2177	0.5685	0.4944	0.032*	
C3	0.4044 (4)	0.43623 (15)	0.6586 (2)	0.0230 (6)	
C4	0.4422 (4)	0.35360 (15)	0.6951 (3)	0.0344 (7)	
H4A	0.5512	0.3517	0.7592	0.052*	
H4B	0.4764	0.3235	0.6349	0.052*	
H4C	0.3236	0.3321	0.7150	0.052*	
C5	0.8772 (4)	0.56384 (16)	1.1123 (2)	0.0232 (6)	
H5	0.8652	0.6160	1.0893	0.028*	
C6	0.9816 (4)	0.53821 (16)	1.2118 (2)	0.0243 (6)	
H6	1.0535	0.5687	1.2698	0.029*	
C7	0.8427 (3)	0.43705 (16)	1.1119 (2)	0.0206 (6)	
C8	0.7814 (4)	0.35502 (14)	1.0811 (2)	0.0318 (7)	
H8A	0.6674	0.3552	1.0198	0.048*	
H8B	0.7466	0.3290	1.1447	0.048*	
H8C	0.8906	0.3277	1.0589	0.048*	
H11	0.192 (5)	0.3164 (18)	0.366 (3)	0.089 (16)*	
H12	0.029 (4)	0.3258 (17)	0.412 (3)	0.052 (11)*	
H21	0.517 (2)	0.2499 (19)	0.374 (3)	0.060 (11)*	
H22	0.392 (5)	0.2426 (19)	0.2737 (10)	0.066 (12)*	

### Atomic displacement parameters (Å^2^)

**Table:**

	*U*^11^	*U*^22^	*U*^33^	*U*^12^	*U*^13^	*U*^23^
Ag1	0.01779 (13)	0.02855 (14)	0.01956 (14)	−0.00075 (8)	0.00074 (9)	−0.00051 (8)
O1	0.0306 (10)	0.0386 (11)	0.0230 (11)	−0.0041 (9)	0.0001 (8)	−0.0002 (8)
O2	0.0438 (12)	0.0364 (12)	0.0350 (13)	−0.0013 (10)	−0.0064 (10)	−0.0126 (10)
O3	0.0549 (14)	0.0491 (13)	0.0362 (13)	−0.0194 (11)	0.0157 (11)	−0.0026 (10)
O1w	0.0270 (11)	0.0253 (11)	0.0268 (11)	0.0008 (9)	0.0038 (9)	0.0015 (8)
O2w	0.0280 (11)	0.0296 (11)	0.0276 (12)	0.0012 (9)	0.0001 (9)	−0.0027 (9)
N1	0.0174 (11)	0.0250 (12)	0.0218 (12)	−0.0016 (8)	0.0017 (9)	−0.0017 (9)
N2	0.0221 (12)	0.0310 (14)	0.0230 (13)	0.0004 (10)	0.0023 (10)	−0.0050 (11)
N3	0.0196 (12)	0.0203 (11)	0.0203 (12)	0.0006 (8)	0.0036 (9)	−0.0008 (8)
N4	0.0223 (12)	0.0226 (12)	0.0204 (12)	0.0018 (10)	0.0037 (10)	0.0034 (10)
N5	0.0212 (11)	0.0294 (12)	0.0305 (14)	0.0031 (10)	0.0019 (10)	−0.0050 (10)
C1	0.0249 (14)	0.0219 (14)	0.0254 (15)	0.0006 (11)	0.0038 (12)	0.0017 (11)
C2	0.0258 (15)	0.0308 (15)	0.0239 (15)	0.0043 (12)	0.0060 (12)	0.0043 (12)
C3	0.0171 (13)	0.0274 (14)	0.0251 (15)	−0.0001 (11)	0.0056 (11)	−0.0034 (11)
C4	0.0351 (16)	0.0227 (15)	0.0426 (18)	0.0058 (12)	0.0008 (14)	−0.0025 (12)
C5	0.0247 (15)	0.0183 (13)	0.0258 (16)	−0.0015 (10)	0.0033 (12)	−0.0043 (11)
C6	0.0223 (14)	0.0264 (15)	0.0247 (15)	−0.0048 (12)	0.0058 (11)	−0.0056 (12)
C7	0.0167 (13)	0.0229 (14)	0.0242 (15)	0.0025 (10)	0.0088 (11)	0.0005 (11)
C8	0.0326 (15)	0.0213 (14)	0.0416 (18)	−0.0033 (12)	0.0075 (13)	−0.0019 (12)

### Geometric parameters (Å, °)

**Table:**

Ag1—N1	2.090 (2)	N4—C6	1.384 (4)
Ag1—N3	2.091 (2)	N4—H4	0.844 (10)
O1—N5	1.260 (3)	C1—C2	1.342 (4)
O2—N5	1.250 (3)	C1—H1	0.9300
O3—N5	1.238 (3)	C2—H2A	0.9300
O1w—H11	0.845 (10)	C3—C4	1.481 (4)
O1w—H12	0.848 (10)	C4—H4A	0.9600
O2w—H21	0.844 (10)	C4—H4B	0.9600
O2w—H22	0.842 (10)	C4—H4C	0.9600
N1—C3	1.326 (3)	C5—C6	1.344 (4)
N1—C1	1.385 (3)	C5—H5	0.9300
N2—C3	1.347 (4)	C6—H6	0.9300
N2—C2	1.377 (4)	C7—C8	1.483 (3)
N2—H2	0.856 (10)	C8—H8A	0.9600
N3—C7	1.323 (3)	C8—H8B	0.9600
N3—C5	1.381 (3)	C8—H8C	0.9600
N4—C7	1.342 (3)		
			
N1—Ag1—N3	178.27 (7)	N1—C3—N2	110.0 (2)
H11—O1w—H12	106 (4)	N1—C3—C4	126.5 (2)
H21—O2w—H22	105 (3)	N2—C3—C4	123.5 (2)
C3—N1—C1	106.2 (2)	C3—C4—H4A	109.5
C3—N1—Ag1	125.87 (18)	C3—C4—H4B	109.5
C1—N1—Ag1	127.32 (17)	H4A—C4—H4B	109.5
C3—N2—C2	108.0 (2)	C3—C4—H4C	109.5
C3—N2—H2	121 (2)	H4A—C4—H4C	109.5
C2—N2—H2	131 (2)	H4B—C4—H4C	109.5
C7—N3—C5	106.7 (2)	C6—C5—N3	109.3 (2)
C7—N3—Ag1	126.03 (17)	C6—C5—H5	125.4
C5—N3—Ag1	126.92 (16)	N3—C5—H5	125.4
C7—N4—C6	108.0 (2)	C5—C6—N4	106.1 (2)
C7—N4—H4	125.1 (18)	C5—C6—H6	127.0
C6—N4—H4	126.9 (19)	N4—C6—H6	127.0
O3—N5—O2	120.2 (2)	N3—C7—N4	109.9 (2)
O3—N5—O1	119.9 (2)	N3—C7—C8	126.5 (2)
O2—N5—O1	119.9 (2)	N4—C7—C8	123.6 (2)
C2—C1—N1	109.5 (2)	C7—C8—H8A	109.5
C2—C1—H1	125.3	C7—C8—H8B	109.5
N1—C1—H1	125.3	H8A—C8—H8B	109.5
C1—C2—N2	106.3 (2)	C7—C8—H8C	109.5
C1—C2—H2A	126.9	H8A—C8—H8C	109.5
N2—C2—H2A	126.9	H8B—C8—H8C	109.5
			
C3—N1—C1—C2	−0.2 (3)	C7—N3—C5—C6	0.1 (3)
Ag1—N1—C1—C2	−171.9 (2)	Ag1—N3—C5—C6	−173.71 (19)
N1—C1—C2—N2	0.4 (3)	N3—C5—C6—N4	0.5 (3)
C3—N2—C2—C1	−0.5 (3)	C7—N4—C6—C5	−1.0 (3)
C1—N1—C3—N2	−0.1 (3)	C5—N3—C7—N4	−0.8 (3)
Ag1—N1—C3—N2	171.74 (19)	Ag1—N3—C7—N4	173.13 (18)
C1—N1—C3—C4	−179.5 (3)	C5—N3—C7—C8	178.6 (2)
Ag1—N1—C3—C4	−7.7 (4)	Ag1—N3—C7—C8	−7.5 (4)
C2—N2—C3—N1	0.4 (3)	C6—N4—C7—N3	1.1 (3)
C2—N2—C3—C4	179.8 (2)	C6—N4—C7—C8	−178.2 (2)

### Hydrogen-bond geometry (Å, °)

**Table:**

*D*—H···*A*	*D*—H	H···*A*	*D*···*A*	*D*—H···*A*
N2—H2···O1w	0.86 (1)	1.99 (1)	2.838 (3)	169 (3)
N4—H4···O1w^i^	0.84 (1)	1.99 (1)	2.837 (3)	178 (3)
O1w—H11···O2w	0.85 (1)	1.89 (1)	2.726 (3)	170 (4)
O1w—H12···O1	0.85 (1)	1.99 (1)	2.826 (3)	171 (3)
O2w—H21···O1^ii^	0.84 (1)	2.02 (1)	2.867 (3)	179 (4)
O2w—H22···O2^iii^	0.84 (1)	2.15 (2)	2.955 (3)	159 (3)

Symmetry codes: (i) *x*+1, *y*, *z*+1; (ii) *x*+1, *y*, *z*; (iii) *x*+1/2, −*y*+1/2, *z*−1/2.
